# On the Use of White Vitriol in Agues

**Published:** 1803-12-01

**Authors:** 

**Affiliations:** Romsey, Hants


### On the Use of White Vitriol in Agues; *communicated by* Mr. Cuming, *of Romsey, Hants.*

I Have long had it in contemplation to publish, through the channel of the Medical Journal, the beneficial effects resulting from the administration of zincum vitriolatum in the cure of intermitting fevers. The indulgencies you have hitherto granted me, demand my acknowledgements, and inspire me with a hope that you will find a place for this Essay.

Bark is the remedy which of all others has long maintained its precedence in this department of physic; but almost every physician of any practice, must have had at some period, convincing proofs of its inefficacy; more particularly particularly on delicate subjects, whose stomachs are naturally irritable: for who has not experienced the nausea and disgust excited from the exhibition of this medicine in such large quantities, as from the symptoms and circumstances of the patient, the case seemed to require; and however partial or prejudiced the practitioner may be with respect to the virtues of this hitherto considered specific, it must be no small satisfaction for him to know, that the Materia Medica contains a remedy equally infallible, and more entitled to the epithet specific. I only ask some of my medical brethren, such as are not acquainted with its virtues, (for there are certainly many who never heard or thought of its powerful effects) to give it a fair trial whenever opportunity may serve. In ships of war, I have often given it under every type of the disease, and never knew it fail. I have had two instances within this month, of its curing two poor women who placed themselves under my care, in circumstances the most unfavourable for any medicines to have the wished-for effect.

One of the patients had suffered from a quotidian to such a degree that her legs became œdematous, her countenance sallow and bloated; to such a cachectic state was she reduced, that she had the appearance of falling an easy prey to dropsy: her stomach was much weakened, and her digestive faculties consequently greatly impaired.

I administer this medicine in the following manner: After the primæ viæ have been thoroughly cleansed by means of an antimonial emetic, I begin by giving the patient two or three grains in the day during the intervals of the fit, in this formula. ℞. pulv. zinci vitriol. gr. ij. vel iij. cons. rosæ, q. s. ft. bol. no. iij. capt. æger. j. 2d. a vel 3tia. quaque hora, ut opus fuerit, et augeatur dosim gr. j. indies. I never had occasion to give more than five grains in the day. When given in the shape of a bolus, I always order it to be washed down with half a cupfull of any proper diluent. The first of the above named patients was cured in three days, the latter in seven.

Give me leave to observe, Gentlemen, if this plan of cure was adopted in hospitals, infirmaries, and other receptacles for the sick; it would not only be a means of saving large quantities of cinchona, but would enable the gentlemen at the head of these departments to appropriate the money set apart for the purchase of this expensive drug to the laudable purpose of supporting the patients' strength, by allowing them a sufficient quantity of ale, porter, or wine.

It is not my intention to depreciate the virtues of bark when properly administered; yet in many cases I am confident it has been productive of incalculable mischief, particularly in fever, where there is a want of secretion. I have known the recovery of patients considerably retarded by giving the decoction too liberally, and have known relapses take place in consequence of it; then, how much more danger is there to be apprehended when taken in substance, where it seldom fails to clog the stomach in such a manner as to destroy all appetite; for if want of appetite in fevers depends upon a deficiency of gastric juice, surely such a remedy will entirely frustrate the intention of the physician.

